# Effective LARC Providers: Moving Beyond Training (Republication)

**DOI:** 10.9745/GHSP-D-16-00258

**Published:** 2016-09-28

**Authors:** James D Shelton, Anne E Burke

**Affiliations:** a Johns Hopkins Bloomberg School of Public Health, Baltimore, MD, USA

## Abstract

Effective and productive providers are the key to successful provision of long-acting reversible contraceptives (LARCs). But LARCs demand more of providers than short-acting resupply methods. In addition to sound training, key elements to developing highly productive providers of LARCs include a thorough understanding of the service delivery system context; selecting providers with the most potential, especially from mid-level cadres; strong mentoring and supportive supervision; and attention to the supply chain and to demand-side support.

Note: This is a republication of an editorial first published in GHSP, Volume 4, Supplement 2, “Long-Acting Reversible Contraception Crucial to Meeting Unmet Need Goals by 2020: Key Papers From the 2016 International Conference on Family Planning.”

The special issue of GHSP, focused on long-acting reversible contraceptives (LARCs) based on papers presented at the 2016 International Conference on Family Planning, provides testimony to the remarkable rise in the popularity of implants and intrauterine devices (IUDs), as well as some limited evidence on permanent methods. As these articles report, we see substantial uptake of LARCs in a wide variety of situations when they are provided in a quality fashion, including provision of a wide choice of methods. These situations include:

- Public and private sectors
- Postpartum
- Postabortion
- Difficult crisis-affected settings
- Peri-urban slums
- With vouchers for those particularly in need
- Via a range of provider cadres.

Moreover, these articles provide insights specifically into the acceptability and provision of IUDs, which have long been recognized as underutilized.

## WHAT IS NEEDED FOR GOOD LARC PROVISION?

For any health service to be successful, going beyond the technology or basic intervention alone is crucial. This lesson is particularly important for LARCs and permanent methods, which are more complicated to provide than short-acting methods. It is essential to address the local context, systems, and infrastructure through which LARCs are provided. Key situational issues include physical resources, staffing, organization of work, and cultural context.

Providers are central to the success of any health program. The global health field tends to focus on training to assure providers’ capabilities. But training alone is not enough. Drawing largely on articles in this issue, we offer observations on some program elements that are necessary to help providers be highly productive in LARC programming.

### Selecting Providers With the Most Potential

Inserting and removing LARCs requires particular skills and more effort than providing many other family planning services, such as pills or injectables. Thus, providers of LARCs must be not only technically competent but also motivated to provide the service again and again. So, one selection criterion is whether a provider is likely to be able to provide LARCs often enough to keep her or his skills and have confidence in them. And that calls for selecting staff who are already motivated to provide, and even be champions for, family planning in general. Also, many nonphysician providers may find satisfaction from “task sharing”—being able to provide a service that was previously reserved for physicians—and so may be enthusiastic about providing LARCs. Providers such as midwives and qualified nurses often suit this role. In addition, it appears crucial to select providers who are likely to remain in their posts for a number of years rather than staff who are going to retire or could be transferred soon.

LARC providers must be not only technically competent but also motivated to provide the service again and again.

### Mentoring and Supervising

It is telling that many of the articles in this special issue that describe full programming efforts emphasize ongoing support to providers, variously referred to as mentoring, supportive supervision, or coaching (Samuel,^1^ White,^2^ Gueye,^3^ Muthamia,^4^ Pleah^5^). A common strategy is to begin by recruiting, training, and deploying a core cadre of mentors, who then train and mentor other providers. Good mentoring greatly benefits any service, providing technical support, accountability, and motivation. But it is especially productive for a potentially challenging intervention such as provision of LARCs, which calls for a higher level of skill, commitment, and motivation, and for which skills are easily lost if not practiced. Good mentoring also provides broader support for provision of all family planning methods, including counseling and practical problem solving.

Good mentoring benefits any service but is especially important for the provision of LARCs.

### Assuring the Supply Chain

A provider without the proper commodities cannot provide the service. And a disrupted supply chain undermines confidence in the entire service. Thus, several of the articles in this issue focus on building and maintaining a reliable supply chain. In addition to the contraceptives themselves, LARC provision requires equipment and supplies such as gloves, antiseptics, and local anesthetics. While external donors may supply the implants and IUDs, especially at the beginning of a project, disposable supplies cannot be overlooked or assumed. A reliable source of ongoing supply is crucial to sustain continuous services, particularly once programs leave the shelter of donor funding.

### Supporting the Demand Side

Several of the articles emphasize demand promotion activities, especially outreach to the community, including religious leaders, and some mass media communication. Promotion of IUDs, in particular, benefits from such activities. Misperceptions about IUDs persist among both providers and the general public, as documented by Twesigye and colleagues.^6^ Addressing such misperceptions is essential. In addition, the use of vouchers can increase demand and service seeking among clients particularly in need, as reported by Boddam-Whetham and colleagues^7^ and Bajracharya and colleagues.^8^

## CONTRAST WITH A LESS SUCCESSFUL PROJECT

The project in Bangladesh reported by Rahman et al.^9^ resulted in some appreciable increase in use but less than in comparison districts, which had no such intensive intervention. The project included substantial training, with some focus on the supply chain and demand-side support. However, it appears the intervention was operating in a much more challenging system context than that in the comparison districts. For example, there were substantially more provider vacancies and fewer clients were contacted by community health workers in the program districts than in the comparison districts. The high vacancy rate of providers speaks to the possible importance of a stronger selection process from the outset. Notably, an appreciable ongoing mentoring or supportive supervision activity was absent. Would the Bangladesh project have been more successful had it had included stronger provider selection and mentoring components? Yes, we believe so.

## CONCLUSION

In the context of availability of a wide range of other methods, provision of LARCs can be the linchpin in the effort to attain FP2020 goals to meet the contraceptive needs of millions of people. The global health community must make the necessary investment to foster the skills and motivation of LARC providers and give them sufficient system support to facilitate program success.

Provision of LARCs can be the linchpin in the effort to attain FP2020 goals.
